# Measuring the professional social capital of psychiatrists: adaptation and validation of the Resource Generator for Psychiatrists (RG-Psy)

**DOI:** 10.1192/j.eurpsy.2023.653

**Published:** 2023-07-19

**Authors:** J. Lagreula, V. Lorant, O. Dalleur

**Affiliations:** ^1^ Clinical Pharmacy research group (CLIP), Louvain Drug Research Institute (LDRI); ^2^ Institut de Recherche Santé et Société (IRSS); ^3^Clinical Pharmacy research group (CLIP)/Louvain Drug Research Institute (LDRI), UCLouvain, Bruxelles, Belgium

## Abstract

**Introduction:**

Psychiatrists need access to professional resources to care for their patients. In mental health settings, clinical innovations such as a new therapeutic approach, clinical guidelines or new drugs can diffuse more or less, depending on the social capital of these clinicians. The Resource Generator developed by Snijders & Van Der Gaag (2004) measures access to resources within a social network for the general population. It may therefore not capture access to social capital in the professional field of psychiatry.

**Objectives:**

We aimed to develop and validate the Resource Generator for Psychiatrists and to detect factors influencing the social capital of clinicians.

**Methods:**

The development of the final 11-item questionnaire followed multiple steps. First, the items were selected and adapted by an expert in the sociology of mental health to match the sector of psychiatry. Content validity and detection of important issues or misunderstandings were ensured by cognitive interviews with a panel of 6 clinicians. Each item has a 6-point response scale, rated from 0 to 6. Answers were coded “0” when the respondent did not need a certain resource or it was not applicable to their situation, while answering the closest resource was coded “6”. The online self-completion questionnaire was administered through a link sent by email to all adult psychiatrists and psychiatric residents licensed to work in Belgium. Additional warm contacts were performed for psychiatrists working in ambulatory care. An exploratory factor analysis was conducted. Internal consistency was ensured with Pearson’s correlation, item-total correlation and Cronbach’s alpha. Test-retest reliability was also measured. Multivariable linear regression analysis assessed the association between psychiatrist demographics and the RG-Psy total score.

**Results:**

The Resource Generator for Psychiatrists questionnaire completed by 152 psychiatrists showed a normal distribution with a mean of 32.5 (SD=12), good test-retest reliability (ICC=0.81), and good total Cronbach’s alpha (0.74). Exploratory factor analysis revealed two main subtypes in psychiatrists’ social capital: “attention and access to advice” and “practical assistance, knowledge and expertise”, with Cronbach’s alpha of 0.62 and 0.7 respectively. Clinicians attending institutional seminars (β=5.5221, p=0.013) and working in multidisciplinary settings such as hospitals (β=4.7448, p=0.023) or a mobile team (β=8.7475, p=0.014) were more likely to have higher social capital.

**Conclusions:**

Psychiatrists’ access to professional resources can be reliably measured by a 11-item questionnaire and can be used to test the influence of their professional social capital on different outcomes.

**Disclosure of Interest:**

None Declared

